# Deoxynivalenol Affects Cell Metabolism and Increases Protein Biosynthesis in Intestinal Porcine Epithelial Cells (IPEC-J2): DON Increases Protein Biosynthesis

**DOI:** 10.3390/toxins10110464

**Published:** 2018-11-09

**Authors:** Constanze Nossol, Peter Landgraf, Stefan Kahlert, Michael Oster, Berend Isermann, Daniela C. Dieterich, Klaus Wimmers, Sven Dänicke, Hermann-Josef Rothkötter

**Affiliations:** 1 Medical Faculty, Institute of Anatomy, Otto-von-Guericke University, 39120 Magdeburg, Germany; stefan.kahlert@med.ovgu.de (S.K.); hermann-josef.rothkoetter@med.ovgu.de (H.-J.R.); 2 Institute for Pharmacology and Toxicology, Otto-von-Guericke University, 39120 Magdeburg, Germany; peter.landgraf@med.ovgu.de (P.L.); daniela.dieterich@med.ovgu.de (D.C.D.); 3 Genomics Unit, Leibniz Institute for Farm Animal Biology, 18196 Dummerstorf, Germany; oster@fbn-dummerstorf.de (M.O.); wimmers@fbn-dummerstorf.de (K.W.); 4 Institute of Clinical Chemistry and Pathochemistry, Otto-von-Guericke University, 39120 Magdeburg, Germany; berend.isermann@med.ovgu.de; 5 Center for Behavior Brain Sciences (CBBS), 39120 Magdeburg, Germany; 6 Institute of Animal Nutrition, Friedrich-Loeffler Institute, 38116 Braunschweig, Germany; Sven.Daenicke@fli.de

**Keywords:** microarray analyses, toxins, DON, BONCAT, cell metabolism, oxygen, IPEC-J2

## Abstract

Deoxynivalenol (DON) is a toxin found in cereals as well as in processed products such as pasta, and causes substantial economic losses for stock breeding as it induces vomiting, reduced feeding, and reduced growth rates in piglets. Oxidative phosphorylation, TCA-cycle, transcription, and translation have been hypothesized to be leading pathways that are affected by DON. We used an application of high and low glucose to examine oxidative phosphorylation and anaerobic glycolysis. A change in the metabolic status of IPEC-J2 was observed and confirmed by microarray data. Measurements of oxygen consumption resulted in a significant reduction, if DON attacks from the basolateral. Furthermore, we found a dose-dependent effect with a significant reduction at 2000 ng/mL. In addition, SLC7A11 and PHB, the genes with the highest regulation in our microarray analyses under low glucose supply, were investigated and showed a variable regulation on protein level. Lactate production and glucose consumption was investigated to examine the impact of DON on anaerobic glycolysis and we observed a significant increase in 2000 bl^high^ and a decrease in 2000 ap^high^. Interestingly, both groups as well as 200 bl^high^ showed a significant higher de novo protein synthesis when compared to the control. These results indicate the direct or indirect impact of DON on metabolic pathways in IPEC-J2.

## 1. Introduction

Deoxynivalenol (DON) is a toxin, which is produced by *Fusarium graminearum* and *Fusarium culmorum* in oats, maize, barley, and wheat. In fact, DON is not the most common toxin, but it is found in crops, and used for food and feed production in Europe and North America [1]. Moreover, DON has been detected as a contaminant in processed products including popcorn, pasta, and beer. Schothorst and Jekel detected a concentration of 200 ng/mL (0.675 µM) DON in their beer samples [2]. 

Critically, DON causes substantial economic losses for stock breeding as it induces vomiting, reduced feeding, and reduced growth rates in piglets [3]. However, the mechanisms underlying these toxicological effects are still unclear. DON affects the immune system and gastrointestinal tract via disruption of the intestinal barrier function and regulation of nutrient transporter gene expression although the effects are not consistent [4,5,6,7]. Several studies have shown that DON inhibits barrier function and induces the proinflammatory gene expression of TNFα, IL-6, and IL-1ß. In vitro, it affects membrane integrity depending on dose, cell line, and exposure time [8,9,10,11]. Furthermore, it has been shown that DON is an inhibitor of protein biosynthesis [3] and that it interferes with the peptidyl transferase function of ribosomes, thereby impairing initiation and elongation [12]. In an in vivo study, Dänicke et al. showed significantly reduced protein synthesis (fractional synthesis rate (FSR)) in the kidneys, spleen, and ileum of DON-exposed pigs, but jejunum and jejunal mucosa cells were not affected by DON [13]. 

Deoxinivalenol influences gene expression and therefore different pathways are affected: TCA-cycle, oxidative phosphorylation, transcription, and translation [8]. Deoxynivalenol affects protein biosynthesis due to the activation of ribosomal-associated kinase PKR. The ribosome-associated kinase PKR phosphorylates eIF2α and inhibits translation [14]. Possibly, mitochondria are an important target of DON because oxidative phosphorylation takes place in mitochondria. Mitochondria are the most important tool to convert pyruvate to CO_2_ and H_2_O in oxidative phosphorylation. Anaerobic glycolysis is used under hypoxic conditions or during proliferation, whereas pyruvate is converted to lactate (no mitochondria is used). Glucose is the most essential substrate in cell culture and is used for both oxidative phosphorylation and anaerobic glycolysis, but cells can be forced to use glutamine if glucose is restricted. Mitochondria generate a high amount of ATP due to the respiratory chain, and play an important role in a wide variety of intracellular processes including signal transduction and protein synthesis. Additionally, different studies have shown that trichothecene toxins induce oxidative stress [15,16,17] due to a significantly increased level of reactive oxygen species (ROS) and the depletion of intracellular reduced glutathione (GSH). Mitochondria-generated ROS play an important role in cytochrome C release and other pro-apoptotic proteins, which can trigger caspase activation and apoptosis [17].

Important morphological features indicating an adequate gut epithelial cell culture model are the development of highly prismatic enterocytes as the monolayer, polarized cell growths with a well-defined apical and basolateral cell membrane compartment, microvilli on the apical side, expression of lateral tight junction complexes enabling the epithelial barrier function, and desmosomes and zonula adhaerens between the epithelial cells [18,19]. Furthermore, a metabolic pathway close to the in vivo situation is an important feature. A technical prerequisite is a monolayer support (comparable with the epithelial basement membrane) with pores. Within many various continuous cell lines, IPEC-J2 provides an exceptional option, as it is both originally isolated from newborn piglets and is non-transformed, and not tumor derived. IPEC-J2 shows a morphological and functional similarity to porcine enterocytes. This cell line represents a well-established model for the simulation of the human intestinal barrier [20].

The aim of this study was to establish a culture model where studying oxidative phosphorylation and anaerobic glycolysis was possible. Furthermore, we wanted to demonstrate the impact of DON on both pathways and on de novo protein synthesis.

## 2. Results

### 2.1. Constitutively Regulated Genes Depend on High Or Low Glucose Concentration

**Figure 1:**
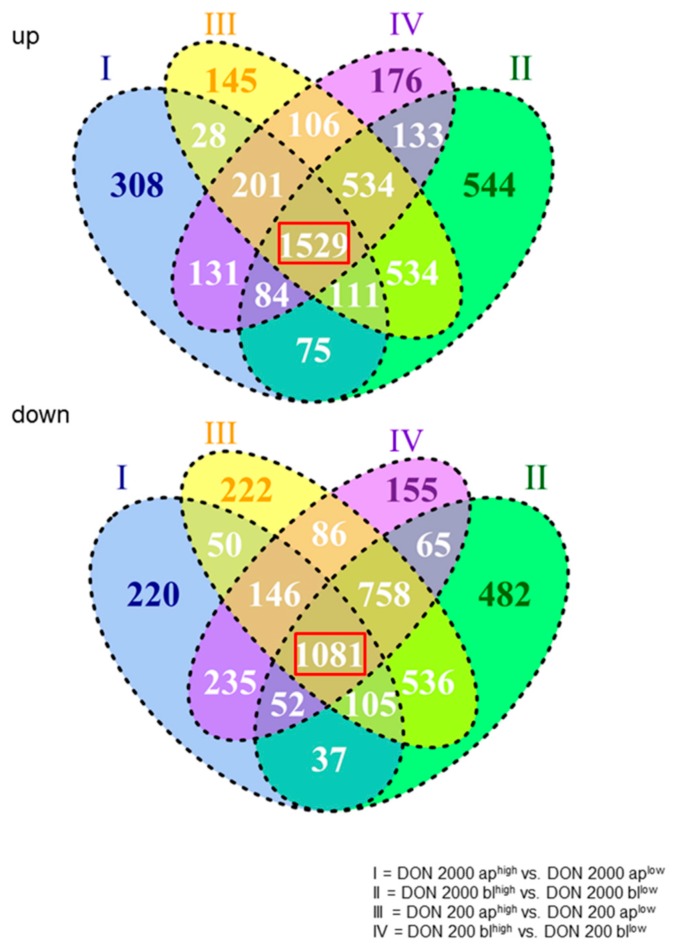
Microarray analyses of deoxynivalenol (DON)-treatment groups dependent on glucose concentration. All significantly regulated genes were analyzed due to a comparison of intersections. A total of 1529 genes were constitutively up-regulated and 1081 were constitutively down-regulated (sum: 2610; red marked; *N* = 3).

**Figure 2:**
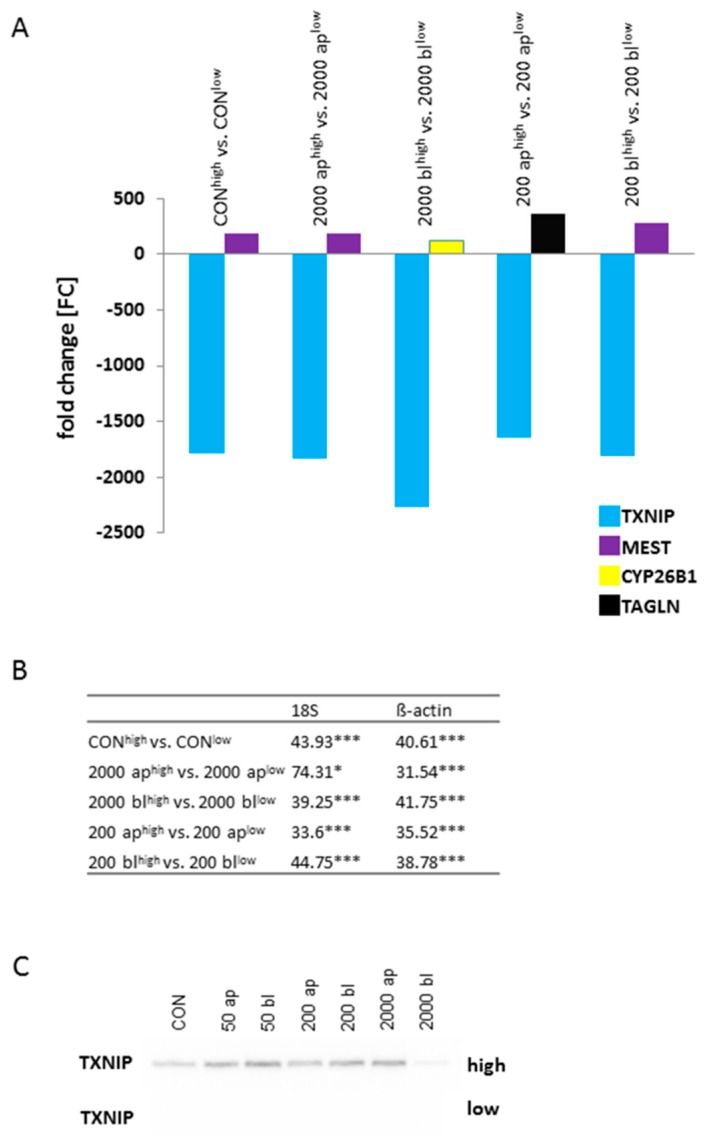
Constitutively regulated genes of the microarray analyses. (**A**) 2610 genes were basically up- or down-regulated in the pairwise comparison of DON-treatment groups dependent on glucose concentration in the microarray analyses. The highest up- and down-regulated genes in the pairwise comparisons within the pool of basically regulated genes are shown: TXNIP (thioredoxin interacting protein), MEST (mesoderm specific transcript), CYP26B1 (cytochrome P450 family 26 subfamily B member 1), and TAGLN (transgelin). (**B**) qPCR data [relative quantification; %] of TXNIP are shown in the table (*N* = 5). All comparisons resulted in a significant down-regulation in DON-treatment groups under low glucose when compared to the appropriate treatment group under high glucose (*p* < 0.05 *; *p* < 0.001 ***). (**C**) A reduction of TXNIP was found on protein level in all DON-treatment groups under low glucose when compared to the equivalent group of high glucose (*N* = 3).

In Figure 1, the comparisons of 2000 ap^high^ vs. 2000 ap^low^, 2000 bl^high^ vs. 2000 bl^low^, 200 ap^high^ vs. 200 ap^low^, and 200 bl^high^ vs. 200 bl^low^ are shown. Overall, 8869 genes were significantly regulated. In the comparison of intersections, we found 2610 genes, which were commonly regulated in all comparisons and named as basically or constitutively regulated genes (red marked in Figure 1). Therefore, 1529 were generally up-regulated and 1081 were down-regulated. In the next step, 2610 genes were used for the analyses with theDatabase for Annotation, Visualization and Integrated Discovery (DAVID) and resulted in the following pathways (first 5): (I) Metabolic pathways, (II) Pathways in cancer, (III) Endocytosis, (IV) PI3K-Akt, and (V) Biogenesis of antibiotics. Metabolic pathways were significantly affected, with the highest number of regulated genes (151; Table 1). With the focus on these 2610 basically regulated genes, four different genes were found in the pairwise comparisons with the highest down- or up-regulation: *TXNIP* (thioredoxin interacting protein), *MEST* (mesoderm-specific transcript homolog protein), *CYP26B1* (cytochrome P450 26B1), and *TAGLN* (transgelin) (Figure 2A). *TXNIP* was found to be down-regulated in all treatment groups^low^ when compared to high glucose (Figure 2A). Furthermore, the data of the microarray analyses were confirmed by qPCR (Figure 2B), and we observed a significant down-regulation in all treatment groups of low glucose when compared to the appropriate treatment group under high glucose. In our Western blot analyses, no TXNIP protein was found in all treatment groups^low^ as well as in the control^low^ cells (Figure 2C) under low glucose conditions. In addition, we analyzed our data by qPCR. Furthermore, a significant up-regulation of TXNIP when compared to control was observed at 2000 ap independent of glucose concentration (Table 2). Under high glucose conditions, we detected a strong expression at 2000 ap when compared to the control, but not under low glucose conditions (Figure 2C). 

### 2.2. General Effect of DON on Gene Expression under Low Glucose Consumption

**Figure 3:**
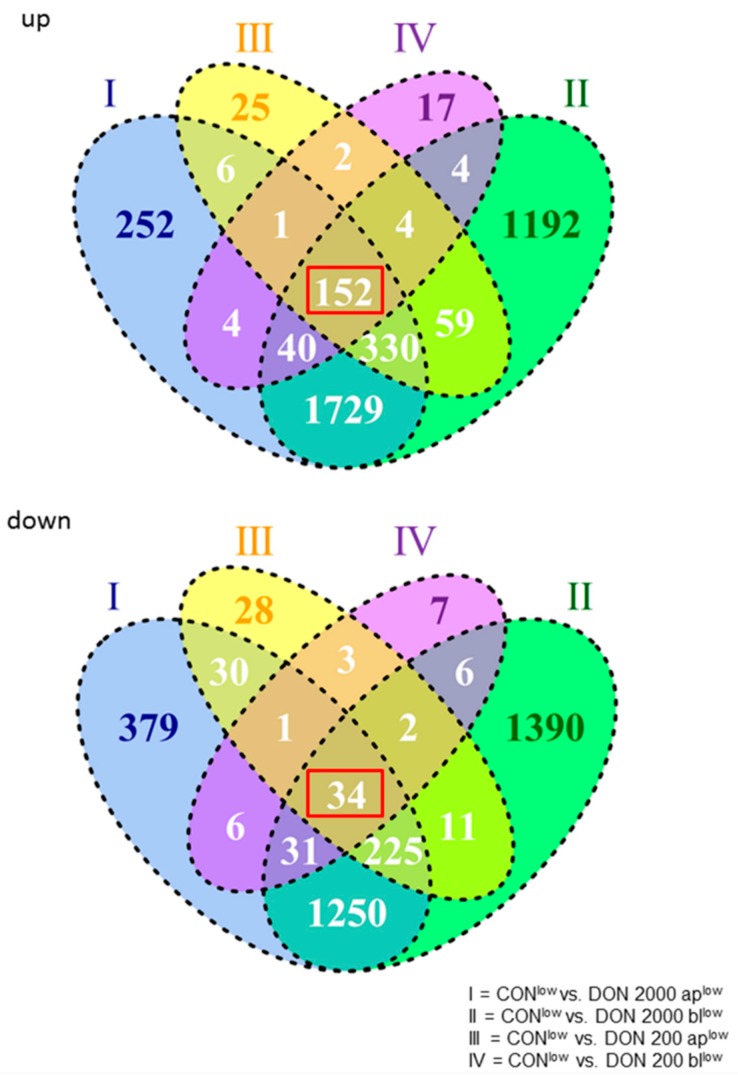
Microarray analyses of DON-treatment groups in comparison to the control under low glucose conditions. A total of 3817 genes were significantly regulated. With the focus on basically regulated genes, we found 152 genes that were up-regulated and 34 genes that were down-regulated (red marked; *N* = 3).

In the IPEC-J2 microarray analyses, 3817 genes were significantly up-regulated and 3412 genes were significantly down-regulated in the comparison of CON^low^ vs. 2000 ap^low^, CON^low^ vs. 2000 bl^low^, CON^low^ vs. 200 ap^low^, and CON^low^ vs. 200 bl^low^. In Figure 3, the intersections of the up-regulated as well as all of the down-regulated genes are shown between the different comparisons. There were 152 genes that were basically up-regulated independent of the DON concentration (red marked in Figure 3), whereas 34 genes were generally down-regulated (red marked in Figure 3). These genes were significantly regulated on principle with DON-impact. Significantly regulated genes (186) were analyzed with DAVID and resulted in only five KEGG-pathways: (I) Spliceosome, (II) RNA transport, (III) Epstein-Barr virus infection, (IV) Ribosome biogenesis in eukaroytes, and (V) mRNA surveillance pathway (Table 3).

**Figure 4:**
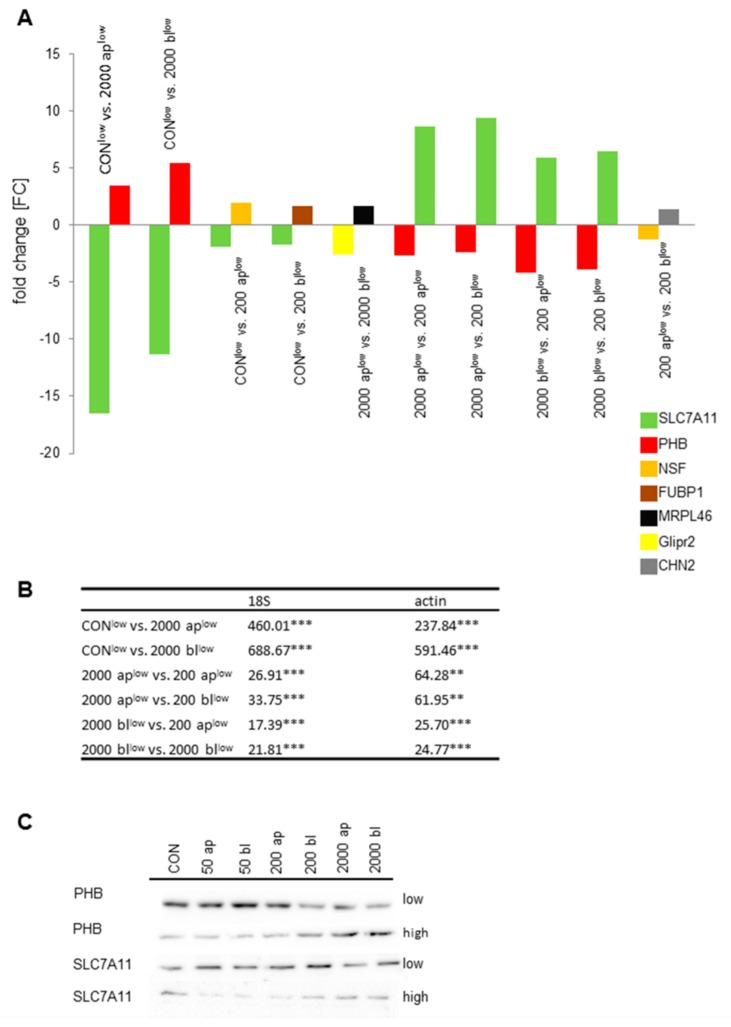
Basic regulated genes. A total of 186 genes were basically up- or down-regulated in the pairwise comparison of the DON-treatment group under low glucose concentration. The highest up- and down-regulated genes in the pairwise comparisons within the pool of basically regulated genes are shown (**A**). SLC7A11 (glutamate transporter) and PHB (Prohibitin) were the most frequently occurring genes in the comparisons. Therefore, SLC7A11 and PHB were analyzed via qPCR (relative quantification [%]; *N* = 5). Comparisons that were significantly regulated were asterisked (*p* < 0.01 **, *p* < 0.001 ***) (**B**), and Western blot (*N* = 3) analysis was performed (**C**). PBH showed a contrarian gradient in the expression depending on glucose consumption, DON-concentration, and application. SLC7A11 had a lower expression in DON 50 ap^high^, 50 bl^high^, and 200 ap^high^ when compared to DON 50 ap^low^, 50 bl^low^, and 200 ap^low^.

In the next step, we looked for the highest up- and down-regulated genes within the pool of basically regulated genes (186) in the pairwise comparisons of the microarray analyses (Figure 4A). SLC7A11 and PHB were the most common, which were significantly regulated. SLC7A11 was significantly down-regulated in the treatment groups of 2000 ap and bl when compared to the control. This was confirmed by qPCR (18S: Kruskal-Wallis; *p* < 0.001; Mann–Whitney Test; Figure 4B). Furthermore, we analyzed our data with a second housekeeper of β-actin and observed the same results (Kruskal–Wallis, *p* < 0.001; Mann–Whitney Test). In addition, 200 ap and 200 bl also showed a significant decrease in the microarray analyses, but not in qPCR (Figure 4B). In addition, we also examined SLC7A11 under high glucose consumption and found a significant reduction of the expression under 2000 ap, but not in 2000 bl (18S: ANOVA, F (6;96) = 4.811; *p* < 0.001; Table 2). The same result was detected using ß-actin (Kruskal–Wallis Test, *p* < 0.001; Mann–Whitney Test).

PHB expression was tested due to qPCR and showed a significant up-regulation in the microarray data (Figure 4A) as well as in qPCR in the comparison of CON^low^ and 2000 ap^low^ (*p* < 0.001; Mann–Whitney test), respectively CON^low^ and 2000 bl^low^ (*p* < 0.001; Mann–Whitney test) (Kruskal–Wallis test; *p* < 0.001; 18S; Figure 4B). The same results were confirmed by β-actin (Kruskal–Wallis test, *p* < 0.001). In addition, in Western Blot analyses a lower PHB content was found in 200 bl^low^, 2000 ap^low^ and 2000 bl^low^ compared to control (Figure 4C). Furthermore, PHB was generally down-regulated in 200 DON-treatment groups when compared to 2000 DON-treatments groups (Figure 4B). In a further analysis, we investigated the effect of DON under high glucose supply (Table 2). Under high glucose supply, we also observed a significant up-regulation in 2000 ap (*p* < 0.001; Mann–Whitney test) and 2000 bl (*p* < 0.001; Mann–Whitney test) with both housekeeping genes (both: Kruskal-Wallis test; *p* < 0.001; Figure 4B) when compared to the control. 

### 2.3. Effect of Glucose Concentration on Oxygen Consumption

**Figure 5:**
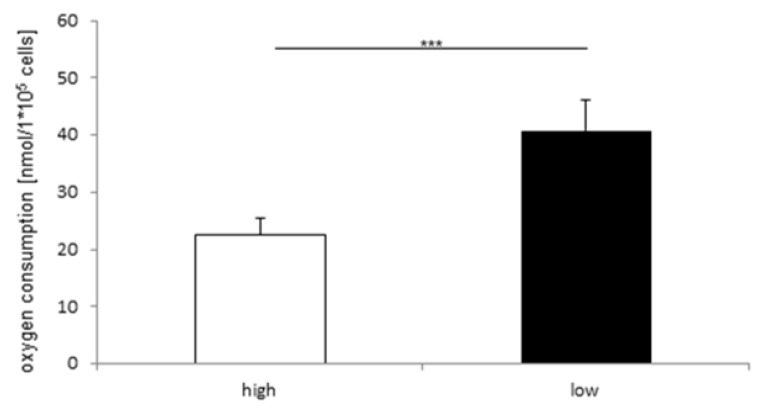
Oxygen consumption under high and low glucose concentration (*N* = 5). Oxygen consumption was measured under high and low glucose conditions. Under low glucose concentration, a higher oxygen consumption was observed than under high glucoses concentration (high: 22.54 nmol/L × 10^5^ cells; low: 40.76 nmol/L × 10^5^ cells, *** *p* < 0.001).

IPEC-J2 was analyzed with the focus on glucose dependent oxygen consumption. IPEC-J2 (40.76 nmol/L × 10^5^ cells) cultured under low glucose conditions showed a significantly higher (2 fold higher; *p* < 0.001) oxygen consumption than the IPEC-J2 growing under high glucose content (22.54 nmol/L × 10^5^ cells; Figure 5).

### 2.4. Influence of DON-Application on Oxygen Consumption

**Figure 6:**
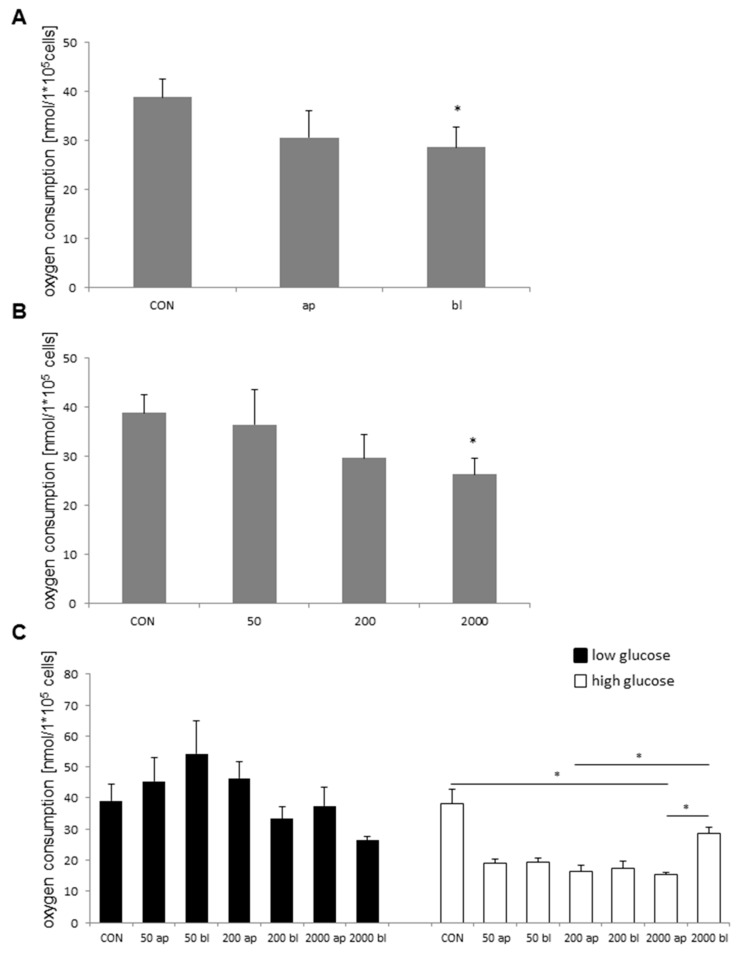
Oxygen consumption (*N* = 5) dependent on DON-application (**A**), concentration (**B**), and glucose concentration (**C**). (**A**) Apical and basolateral application independent of glucose concentration and DON-concentration resulted in a slight, but not significant decrease in apical application when compared to the control (Kruskal–Wallis Test; *p* = 0.01; Mann–Whitney Test; *p* = 0.003); control: 38.85 nmol/L × 10^5^ cells; apical: 30.60 nmol/L × 10^5^ cells) and showed a significant decrease in basolateral application (28.68 nmol/L × 10^5^ cells). (**B**) In the next step, the effect of different DON concentrations on oxygen consumption was analyzed. Therefore, all data were summarized and divided into four groups (Kruskal–Wallis Test; *p* = 0.019; Mann–Whitney Test; *p* = 0.006; CON: 38.85 nmol/L × 10^5^ cells; 50: 36.44 nmol/L × 10^5^ cells; 200: 29.66 nmol/L × 10^5^ cells; 2000: 26.34 nmol/L × 10^5^ cells). A significant difference was found between CON vs. 2000. (**C**) Under low glucose supply, no significant differences were detectable (Kruskal–Wallis; *p* = 0.089): CON 38.85 nmol/L × 10^5^ cells; 50 ap: 45.42 nmol/L × 10^5^; 50 bl: 54.34 nmol/L × 10^5^; 200 ap: 46.30 nmol/L × 10^5^; 200 bl: 33.24 nmol/L × 10^5^; 2000 ap: 37.34 nmol/L × 10^5^; 2000 bl: 26.37 nmol/L × 10^5^ cells. Furthermore, oxygen consumption under different DON concentrations and high glucose supply was analyzed (Welch ANOVA; *p* = 0.004). A trend of lower oxygen consumption was detected under 50 ap (19.16 nmol/L × 10^5^ cells; *p* = 0.089), 50 bl (19.23 nmol/L × 10^5^; *p* = 0.088), 200 ap (16.4 nmol/L × 10^5^ cells; *p* = 0.051), and 200 bl (17.46 nmol/L × 10^5^ cells; *p* = 0.062). A significant difference was found between CON vs. 2000 ap (15.22 nmol/L × 10^5^ cells; *p* = 0.047), 200 ap vs. 2000 bl (28.47 nmol/L × 10^5^ cells; *p* = 0.035), and 2000 ap vs 2000 bl (*p* = 0.016). (*p* < 0.05 *)

To analyze the possible effect of the application side, all data were sorted into the control, apical, and basolateral. This resulted in a slight, but not significant, decrease of the apical when compared to the control (control: 38.85 nmol/L × 10^5^ cells; apical: 30.60 nmol/L × 10^5^ cells) and showed a significant reduction of the basolateral when compared to the control (basolateral: 28.68 nmol/L × 10^5^ cells; Figure 6A).

### 2.5. Oxygen Consumption and DON-Concentration

Furthermore, we examined the effect of DON concentration on oxygen consumption. A significant decrease was detected in the comparison of the control (38.85 nmol/L × 10^5^ cells) and DON 2000 (26.34 nmol/L × 10^5^; Kruskal–Wallis test, *p* = 0.01; Mann–Whitney test, *p* = 0.003; Figure 6B). Additionally, we tested different DON concentrations combined with the two different applications of apical and basolateral. No significant differences were detected under low glucose, but a marked increase at 50 bl (Figure 6C). Under high glucose (Welch ANOVA, *p* = 0.004; Games Howell; Figure 6C), a trend of reduced oxygen consumption was observed in the comparisons between the control and DON-treatment groups: 50 ap (19.16 nmol/L × 10^5^ cells; *p* = 0.089), 50 bl (19.23 nmol/L × 10^5^ cells; *p* = 0.088), 200 ap (16.4 nmol/L × 10^5^ cells; *p* = 0.051), and 200 bl (17.46 nmol/L × 10^5^; *p* = 0.062). A significant difference was observed between CON vs. 2000 ap (*p* = 0.047), 200 ap vs. 2000 bl (*p* = 0.035), and 2000 ap and 2000 bl (*p* = 0.016)

### 2.6. Glucose Consumption and Lactate Production under High Glucose Concentration

**Figure 7:**
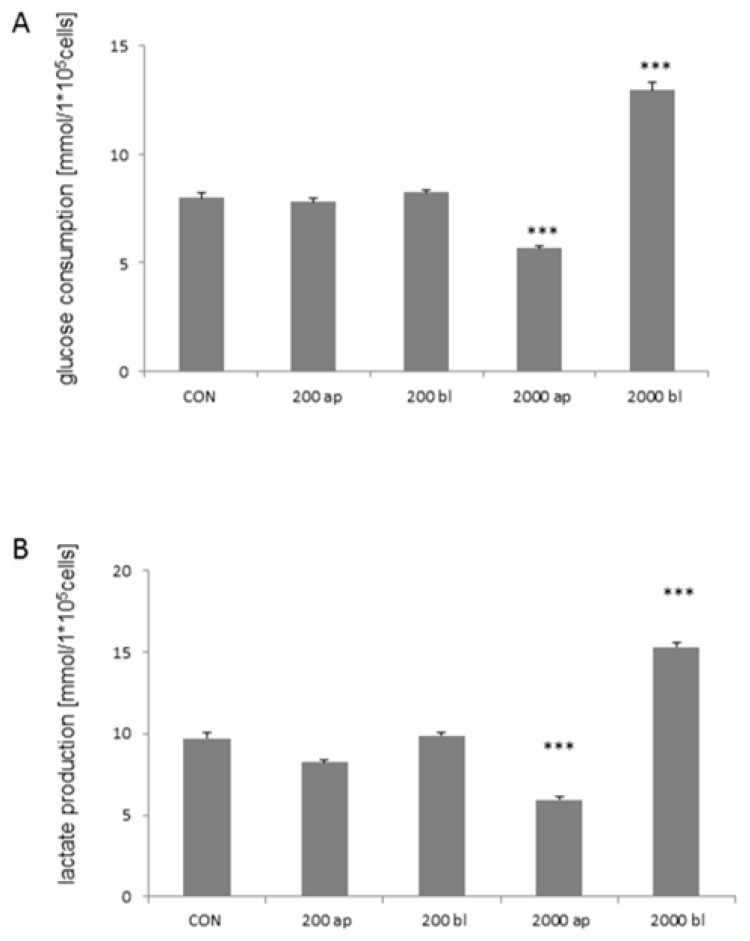
Glucose consumption and lactate production (*N* = 5). (**A**) Glucose consumption was measured in the apical and basolateral compartments. The overall consumption was measured and analyzed. In the DON treatment groups 2000 ap and 2000 bl, significant differences when compared to all other treatment groups could be detected (Welch ANOVA; Games Howell, *p* < 0.001). (**B**) Lactate production was analyzed in the apical and basolateral compartments and overall production was calculated (ANOVA; F (4;28) = 183.068; *p* < 0.001). A significantly higher production was found in 2000 bl (15.32 mmol/L × 10^5^ cells). A lower production was found in 2000 ap (5.96 mmol/L × 10^5^ cells; *p* < 0.001). Furthermore, significant differences were detected in all comparisons against each other with the exception of CON vs. 200 bl. (*p* < 0.001 ***).

Furthermore, glucose consumption was analyzed in the apical and basolateral supernatants. Overall consumption was averaged and we found a significantly higher glucose consumption in 2000 bl (12.94 mmol/L × 10^5^ cells; Welch ANOVA; *p* < 0.001; Games Howell; Figure 7A) when compared to all other treatment groups. On the other hand, we detected a significantly lower glucose consumption in 2000 ap when compared to all other groups (5.66 mmol/L × 10^5^ cells; *p* < 0.001). 

In addition, the lactate production of the cells was measured, and we observed similar results as shown in glucose consumption. A significantly higher production was found in 2000 bl (15.32 mmol/L × 10^5^ cells; *p* < 0.001; Figure 7B) and a lower production in 2000 ap (5.96 mmol/L × 10^5^ cells; *p* < 0.001) when compared to all other treatment groups (ANOVA; F (4;28) = 183.068; *p* < 0.001). Furthermore, significant differences were detected in the comparisons of all with each other except for CON vs. 200 bl. In addition, important genes of glucose- and lactate transport were analyzed via qPCR. A significant increase in GLUT1-RNA-level was found in 2000 ap and bl independent of glucose concentration or the used housekeeper (Table 2; *p* < 0.001). A marked up-regulation was also found in all other DON-treatment groups^low^ when compared to the control. An increased SGLT1-RNA-level was detected in 50 ap and 50 bl under low glucose concentrations, but was decreased in 2000 bl (*p* < 0.001; β-actin). Decreasing values were also found with the second housekeeper 18S. Furthermore, MCT1 was analyzed using qPCR. We observed an up-regulation at 50 ap, 50 bl, and 2000 bl (Table 2; *p* < 0.001, β-actin). A marked, but not significant, increase was also found using 18S as the housekeeper.

### 2.7. Metabolic Pathway in IPEC-J2

**Figure 8:**
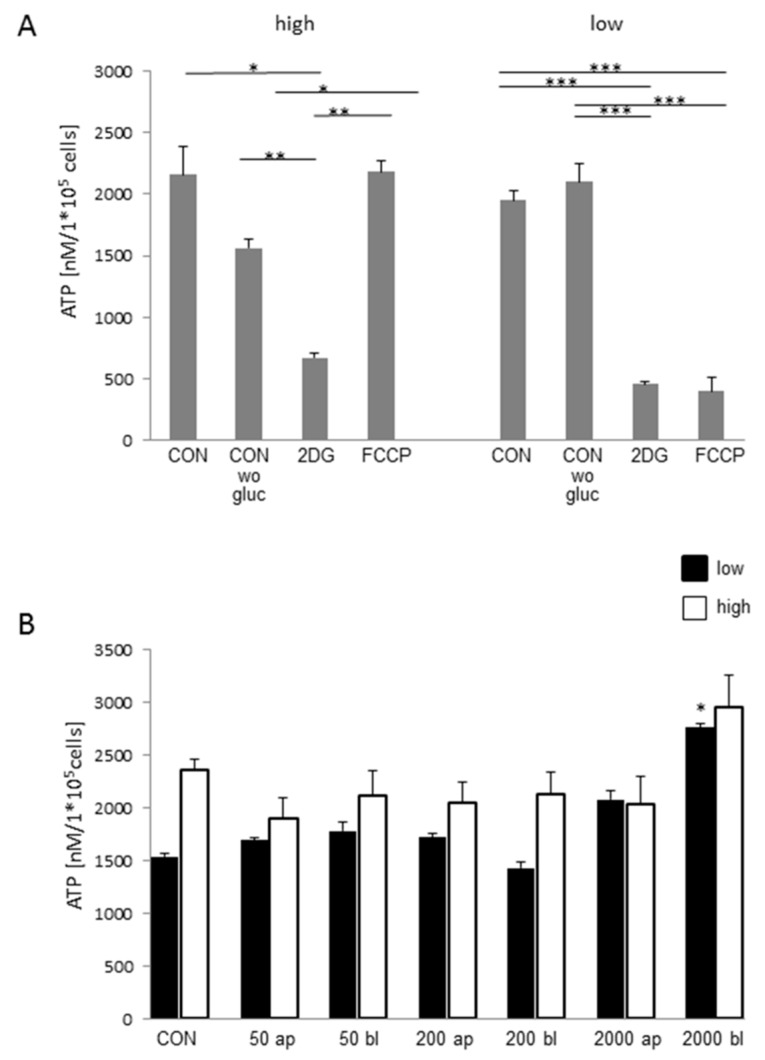
ATP-content (*N* = 3). (**A**) ATP values for high glucose conditions were analyzed by Welch ANOVA (*p* < 0.001; Games Howell. A significantly lower ATP concentration was found in the treatment group of 2DG when compared to the control (*p* = 0.036). 2DG (*p* = 0.0002) and FCCP (*p* = 0.012) were significantly different from CON wo gluc. Additionally, FCCP (*p* = 0.002) showed a significantly higher ATP content than 2DG. Under low glucose supply (ANOVA F (3,8) = 61.463; *p* < 0.001), 2DG and FCCP showed a significantly lower ATP content when compared to the control. The same result was observed when compared to CON wo glucose. (**B**) No differences were observed with the focus on ATP concentration under high glucose conditions (Kruskal–Wallis Test; *p* = 0.653). Furthermore, 2000 bl showed a significantly higher ATP concentration under low glucose supply when compared to all other treatment groups (ANOVA F (6;14) = 11.768; *p* < 0.001; Tukey). Additionally, 200 bl was significantly lower when compared to 2000 ap (*p* = 0.040). In the comparisons of high and low glucose with the appropriate DON-treatment, no differences were detected. (*p* < 0.05 *; *p* < 0.01 **; *p* < 0.005 ***).

Figure 8A shows the ATP analyses under different treatments to evaluate the metabolic pathway of IPEC-J2 under high and low glucose conditions. Under high glucose conditions, we detected no significant decrease of ATP in the treatment group without (wo) glucose when compared to the control and FCCP when compared to the control. A significant decrease in the ATP content was observed in the cells that were treated with 2DG in comparison to the control and FCCP (Welch ANOVA; *p* < 0.001; Games Howell). In contrast, a significant decrease was found in FCCP and 2DG when compared to the control (ANOVA; F (3;8) = 61.463; *p* < 0.001; Figure 8A) under low glucose conditions.

### 2.8. ATP-Concentration Depends on Glucose- and DON-Concentration

Under high glucose conditions (Figure 8B), we observed no differences in the comparison of DON-treatment groups to the control (Kruskal–Wallis Test; *p* = 0.653). In the next step, we analyzed the treatment groups under low glucose supply and found a significantly higher ATP concentration in the 2000 bl DON-treatment group when compared to all other treatment groups (ANOVA F (6;14) = 11.768; *p* < 0.001; Tukey). In the comparisons of high and low glucose with the appropriate DON-treatment, we detected no differences. 

Furthermore, COX5B is an important gene of the respiratory chain, which encodes cytochrome C oxidase subunit 5B. Cytochrome C oxidase is the terminal enzyme of the last step of the respiratory pathway in the mitochondria. A significant upregulation of COX5B was found in 2000 ap independent of glucose concentration (Table 2; *p* < 0.001; β-actin) and was confirmed by 18S for 2000 ap^low^ (*p* < 0.001). In 2000 ap^high^, a marked but not significant increase was found with 18S.

### 2.9. Protein Biosynthesis Rate Depend on DON-Concentration and Application under High Glucose Conditions

**Figure 9:**
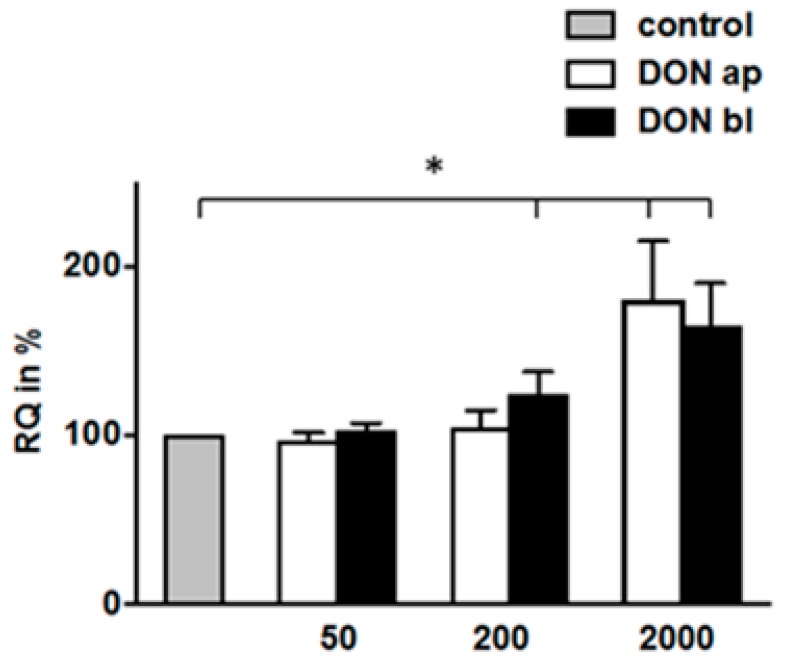
Analyses of protein biosynthesis under high glucose concentration (*N* = 3). After 72 h of DON treatment, cells were handled as described above. A significantly higher protein synthesis rate (RQ: relative quantification in %) was found in the 200 ng/mL bl, 2000 ng/mL ap and bl treatment groups. (*p* < 0.05 *).

After 72 h of DON-incubation, we detected increasing synthesis rates starting from 200 ng/mL and marked higher protein biosynthesis in the 2000 ng/mL ap and bl treatment groups when compared to the control (Figure 9). In contrast, the protein synthesis rates in groups treated with 50 ng/mL DON remained unaffected.

## 3. Discussion

In our study, we used two different glucose concentrations to trigger the two different pathways, anaerobic glycolysis and oxidative phosphorylation, in IPEC-J2 to address the question of whether DON had an impact on metabolic function. IPEC-J2 uses glycolysis to a higher proportion as shown by the hexokinase inhibitor 2DG and the uncoupler FCCP [21]. Glycolysis is characterized by the release of lactate into the medium and is detectable as the extracellular acidification rate (ECAR) and lower efficiency of ATP syntheses [21,22]. We also detected a significantly decreased ATP level under high and low glucose conditions in our cells with the use of 2DG, indicating glucose as the principle energy source in IPEC-J2 cultures. As a result, the uncoupling of ATP generation by FCCP reduced cellular ATP content only in low glucose conditions. Similar to this is the increased oxygen consumption under low glucose conditions, indicating the optimized usage of glucose derived pyruvate in oxidative phosphorylation. In the microarray analyses of high and low glucose combined with different DON-concentration, we detected different pathways which were significantly regulated: (I) Metabolic pathways, (II) Pathways in cancer, (III) Endocytosis, (IV) PI3K-Akt signaling pathway, and (V) Biosynthesis of antibiotics. Metabolic pathways were the pathway with the highest number of regulated genes. This confirmed our examination model of using high and low glucose to trigger different metabolic pathways in our cells. TXNIP (thioredoxin-interacting protein) was regulated to the highest degree. TXNIP, also known as VDUP1 (vitamin D3 upregulated protein1), is a negative regulator of thioredoxin (TRX) [23], which is an intracellular thiol reductant, and an important regulator of redox balance [24]. Furthermore, TXNIP is an important regulator of cellular glucose and fatty acid metabolism [25], and its transcription is highly induced by glucose [26]. A high expression of the TXNIP protein resulted in reduced glucose uptake and vice versa, a reduced TXNIP protein induced an up-regulation of glucose uptake [27]. We found a down-regulation of TXNIP in our array data in all treatment groups^low^ when compared to all groups with high glucose. This was confirmed by Western blot analyses. We detected no TXNIP protein in any of the treatment groups^low^. This makes sense as cells want to up-regulate glucose uptake in a glucose-restricted environment. Furthermore, we found a down-regulation of the TXNIP protein in DON 2000 bl^high^ when compared to all other treatment groups^high^, which agreed with the data of the glucose consumption of the cells. Here, we found a significantly higher value for 2000 bl. Another interesting gene is transgelin (TAGLN), which was up-regulated in our examination in the treatment group DON 200 bl^low^ when compared to DON 200 bl^high^. TAGLN is a shape-change sensitive actin-binding protein of the calponin family and is found in the cytoskeletal apparatus [28], such as, for example, in the smooth muscle tissue of normal adult vertebrates [29]. Recent studies have provided further evidence that transgelin is also both a tumor suppressor and a variable tumor biomarker, which depends on the tumor type, stage, and experimental model [30,31].

Diesing et al. analyzed the impact of DON under high glucose concentration. They detected TRA1 and CAV2 in response to apical and basolateral DON application (200 and 2000 ng/mL) [9]. TRA1 mRNA was generally down-regulated in the microarray and CAV2 up-regulated. TRA1 is also known as GRP94 or endoplasmin. The promoters of the grp genes constitutively express their gene products, and their promoter activities can be further enhanced in cellular environments of low glucose or oxygen [32]. Awad and Zentek observed an effect of DON, which acts on the intestinal sugar transport by reducing the synthesis of transporter carriers [33]. We also indirectly observed an impact on the glucose transporter as a result of measuring glucose consumption in the apical and basolateral medium, whereas 2000 ap resulted in the lowest and 2000 bl in the highest glucose consumption. This was in agreement with our TXNIP protein data. A down-regulation of TXNIP, which agreed with a higher degree of glucose uptake, was found in 2000 bl^high^. In 2000 ap^high^, we found the same results described by Awad and Zentek, where there was a significant decrease in glucose consumption followed by unaffected TXNIP-protein in our analyses. Additionally, we detected decreased lactate production in 2000 bl^high^, a significantly reduced oxygen consumption, and a significantly up-regulated de novo protein synthesis combined with a marked increase of ATP. Diesing et al. showed in their work, with the focus on an intact intestinal barrier, no or only a weak effect on ZO-1 and Claudin-3, which are two important proteins of the tight junction. They found a stable TEER at 2000 ap, but decreased values for 2000 bl [8]. Furthermore, they detected a reduced viability. Our analyses resulted in the same observation of decreased TEER values (Supplementary Materials). Therefore, we assumed that there was an intact barrier in 2000 ap and that the transporter was influenced because of 2000 ap. In contrast, 2000 bl led to a disruption of the intestinal barrier combined with an influence on cell cycle. Diesing et al also found a significantly higher number of BrdU-positive cells in 2000 bl in comparison to the control at 48 h and 72 h of DON-incubation [8]. BrdU is used to label proliferating cells. There is a need for glucose in proliferating cells, which cannot be replaced by other metabolizable substances [34]. We hypothesized that a high BrdU-rate, an up-regulation of SLC7A11, higher glucose uptake, high lactate production, up-regulation of de novo protein synthesis, increased ATP content, and possibly also a higher PHB protein content were involved in an activation of proliferation which acted as a survival strategy in 2000 bl^high^. In contrast, Dänicke et al. could not show any differences in the protein synthesis (fraction synthesis rate; FSR) in the jejunum or jejunal mucosal cells when compared to the control group, but there was a significant reduction in the ileum [13]. SLC7A11 and PHB were the highest up- or down-regulated genes in the comparisons of low glucose. Glutamate/cysteine antiporter solute carrier family 7 member 11 (SLC7A11) was down-regulated in all treatment groups when compared to the control. This was confirmed by qPCR in 2000 ap and 2000 bl. SLC7A11 is a Na^+^ independent anionic amino acid transport system, which is highly specific for cysteine and glutamate, that imports extracellular cysteine and exports intracellular glutamate [35]. The uptake of cystine into the cell leads to a rapid reduction to cysteine, the rate limiting amino acid for GSH biosynthesis [36]. GSH in turn protects against the cellular damage caused by free radicals and other reactive oxygen species (ROS) such as superoxide and H_2_O_2_ [37]. With the focus on the SLC7A11 protein, we only found weak differences between the treatment groups but under low glucose conditions, a marked down-regulation under 50 ap, 50 bl, and 200 ap when compared to the other treatment groups. It is known that DON induces ROS generation [38]. Increased ROS production leads to oxidative stress that affects endothelial and vascular function, and contributes to vascular disease [39]. An up- or down-regulation of SLC7A11 has a vital influence on the response to oxidative stress induced by DON because SLC7A11 delivers the required cystine for GSH biosynthesis, which protects against oxidative stress. An up-regulation of the SLC7A11 protein under high glucose in the DON-treatment group may be a reply to induced ROS production to enhance GSH production. Differences between low and high glucose with a focus on SLC7A11 expression in general can be induced by the low glucose consumption of the cells and increased ATP demand at the same time. Therefore, cells will increasingly use glutamine as an energy source [40]. Glutamine metabolism results in the generation of key stimulus secretion coupling factors including glutamate and glutathione, which indirectly stimulate ATP production and enhance insulin secretion [39]. Very high concentrations of L-glutamine were used in cell culture (2 up to 10 mM), but physiological concentrations were lower 0.7 mM [41]. We supposed that DON comes into action in glutamine metabolism and influences the glutamine uptake, and on the other hand, indirectly on glutamate efflux due to the SLC7A11 transporter of the cells.

Oxygen is necessary for mitochondrial oxidative phosphorylation. Most mammalian cells in culture predominantly use two sources for energy production: glucose and glutamine. These two molecules supply most of the carbon, nitrogen, free energy, and reducing equivalents necessary for cell growth and division [42]. A previous study showed that 90% of glucose and 60% of glutamine is converted into lactate or alanine in glioblastoma cells in culture [43]. Both lactate and alanine are secreted from the cell as waste, but more important is the robust generation of NADH, which agrees with the reduction to lactate [44]. 

One of our questions was how DON modulated the metabolic pathway. A significantly lower glucose consumption was found in DON 2000 ap, but was significantly higher in 2000 bl, which also resulted in a significantly lower lactate production in 2000 ap but was significantly higher in 2000 ng/mL bl. In vivo, Bannert et al. showed a lower glucose level at the portal sampling site at 30–45 min in DON-fed pigs (4.59 mg DON/kg feed) when compared to the control group, but did not detect any increase or decrease in the lactate levels in pigs in the comparison of CON-fed and DON-fed control groups in their study [45]. 

The second most significantly regulated gene in all comparisons in our microarray analysis under low glucose supply was PHB. PHB is also known as B-cell-receptor-associated protein 32 (BAP 32). It is an evolutionary conserved protein that is found in eukaryotic organisms [46] and occurs in normal cell membranes; moreover, it is an important protein of mitochondria [47]. We found a significantly up-regulated PHB gene at 2000 ng/mL independent of glucose concentration, but had a differential regulation under high or low glucose consumption. PHB1 and PHB2 are two related proteins that always form a complex in the inner membrane of the mitochondria [48] and regulate cell survival [49], proliferation, and energy metabolism [50]. Under high glucose, we detected an up-regulation and under low glucose supply, there was a down-regulation. Jiang et al. showed that an overexpression of PHB was found in breast cancer tissue and was significantly regulated with tumor recurrence [51]. They also postulated that PHB was one of the major underlying mechanisms of breast cancer development and progression [51]. PHBs have been shown to be associated with complex IV subunits in yeast [52] and with complex I subunits in mammalian cells [42], hinting at the possibility that PHBs might participate in the assembly of mitochondrial electron transport chain (ETC) complexes. Furthermore, Kathiria et al. showed that a deficiency in PHBs led to increased generation of reactive oxygen species (ROS) [48,53], but the underlying mechanism of PHB in mitochondrial ROS production is unknown. With regard to mitochondria, prohibitins also influence the stability of mitochondrial translated proteins [48,54]. The assembly of OxPhos complexes is a complicated process and there is a need for both gene products from nuclear and mitochondrial genome. In COX, the subunits are arranged as stoichiometric amounts. Nuclear and mitochondrial encoded subunits need to be provided in a one-to-one ratio [48]. In the metabolic switch between aerobic and anaerobic metabolism, there is a possibility of a temporary imbalance between nuclear and mitochondrial gene products. This occurrence of unassembled, hydrophobic mitochondrial translation products in the inner mitochondrial membrane can cause proton leakage. Prohibitin can also function as a holding complex and to prevent them from misfolding [48].

## 4. Conclusions

Overall, our results showed that DON had an effect on oxygen consumption if it attacks from the basolateral side (Figure 6A). Furthermore, we detected a concentration-dependent impact with the highest effect at 2000 (Figure 6B). Furthermore, we detected differences in glucose consumption and lactate production in 2000 ap^high^ and 2000 bl^high^ when compared to the control. If DON attacks from the basolateral side at 2000, we supposed that DON activated the cell cycle (proliferation) as a survival strategy and therefore, lactate production, glucose uptake, and de novo protein synthesis was up-regulated. Contrary results were found if DON operated from the apical side at 2000 because it resulted in significant higher de novo protein production. Important genes such as COX5B, SLC7A11, and PHB were significantly regulated independent of glucose under high DON-concentration. We assumed that DON directly or indirectly affects cell metabolism due to decreased glucose consumption and lactate production in 2000 ap^high^. This was supported through higher oxygen consumption under 50 ng/mL DON. Further investigations must to be performed with the focus on de novo protein synthesis, lactate production, and glucose consumption under low glucose conditions.

## 5. Materials and Methods

### 5.1. Cell Culture

Intestinal porcine epithelial cells (a friendly gift from Mariana Roselli, Rome, Italy; IPEC-J2 ACC 701; [55,56,57]) were regularly tested and found to be free of mycoplasma contamination (Venor GeM Mycoplasma Detection Kit; Minerva Biolabs, Berlin, Germany). DMEM F12 (1:1) (with glutamine: 2.5 mM, with D-glucose: 3.16 g/L) supplemented with 5% fetal bovine serum (FBS), 16 mM 4-(2-hydroxyethyl)-1-piperazineethansulfonic acid (HEPES), 1% insulin-transferrin-selenium (ITS) (all from PAN-Biotech, Aidenbach, Germany), and 5 ng/mL epidermal growth factor (EGF, Biochrome, Berlin, Germany) was used as cell culture medium (glucose concentration: 3.16 g/L; [high]). Medium with low glucose concentration was made with the same supplementations but DMEM/HAMs F12 was mixed with glucose-free DMEM 1× (with glutamine 4 mM, without d-glucose; Gibco, Carlsbad, USA) to reach an end concentration of 0.03 g/L glucose [low]. In all experiments, cells were seeded with a density of 0.88 × 10^5^/cm^2^ on permeable supports (ThinCerts; culture size: 113.1 mm^2^; pore size: 1 µm; polyester, Greiner bio-one, Frickenhausen, Germany) in media with high or low glucose concentration (high or low). In the apical compartment, 1 mL was used and in the basolateral compartment, we used a 2 mL volume. The medium was changed on days 2, 4, and on day 7, it was supplemented with DON with no further change. Cells grew at 39 °C in an atmosphere of 5% CO_2_ and 100% relative humidity. The transepithelial electrical resistance was measured on days 7, 8, 9, and 10 of cell culture using a Millicell-TERS-electrode (Millipore, Berlin, Germany; Figure S1). 

All of the experiments described below were performed under high glucose and low glucose supply with the exception of the BONCAT analyses (only high glucose).

### 5.2. DON-Application

Deoxynivalenol (DON; D0156; Sigma-Aldrich, Taufkirchen, Germany) was dissolved in absolute ethanol (99.6%; Roth, Germany; end concentration of ethanol 1%) to a 0.2 mg/mL stock solution and a working dilution was prepared in the cell culture medium. Two low DON concentrations of 50 ng/mL and 200 ng/mL and a high DON concentration of 2000 ng/mL were applied, reflecting a non-toxic and a toxic dosage as found in conventional toxicological studies [8]. There were two different application sides: apical or basolateral. This resulted in the following treatment groups: CON, DON 50 ng/mL ap (50 ap), DON 50 ng/mL bl (50 bl), DON 200 ng/mL ap (200 ap), DON 200 ng/mL bl (200 bl), DON 2000 ng/mL ap (2000 ap), and DON 2000 ng/mL bl (2000 bl). On day 7, the medium was exchanged to a medium including different DON-concentrations and cells were incubated for a further 72 h with DON.

### 5.3. RNA Isolation

After withdrawal of the apical and basolateral medium, cells were covered with TRIzol Reagent (Invitrogen, Germany) as described by the manufacturer’s protocol and scraped off the membrane. After adding chloroform to the cell lysate, the supernatant was extracted and RNA was precipitated using isopropanol alcohol. Using 75% ethanol, the RNA was purified and stored in RNA-free water peqGOLD (PEQLAB, Germany) at –80 °C until further processing. Five independent experiments were performed. The RNA was isolated using TRIzol Reagent as per the manufacturer’s directions (Sigma-Aldrich, Taufkirchen, Germany). RNA integrity was checked using the 2100 Bioanalyzer platform (Agilent). RNA concentrations were measured on a NanoDrop ND-1000 spectrometer (PEQLAB, Erlangen, Germany). The absence of genomic DNA was checked by PCR amplification of the porcine GAPDH gene. All RNA samples were stored at –80 °C until downstream analyses were performed.

### 5.4. Microarray

#### 5.4.1. Target Preparation and Hybridization

Regarding comparison I, individual samples covering four treatment groups (DON 200 ap, DON 200 bl, DON 2000 ap, DON 2000 bl) at either low or high glucose level were used for expression analysis (n = 3 per treatment group and glucose level; n = 24). The outcomes of this were four single comparisons: DON 2000 ap^high^ vs. DON 2000 ap^low^, DON 2000 bl^high^ vs. DON 2000 bl^low^, DON 200 ap^high^ vs. DON 200 ap^low^, and DON 200 bl^high^ vs. DON 200 bl^low^. Comparison II comprised individually treated samples (DON 200 ap, DON 200 bl, DON 2000 ap, DON 2000 bl) and control samples at low glucose level (n = 15) (data shown in Figure 3). For the microarray experiments, individual biotin-labeled cRNA samples were hybridized on Affymetrix GeneChip Porcine Genome Arrays according to the manufacturer’s directions (Affymetrix, Santa Clara, CA, USA). The raw data were deposited in a MIAME compliant database [58], the National Center for Biotechnology Information Gene Expression Omnibus (www.ncbi.nlm.nih.gov/geo; accession numbers: GSE 111184 and GSE 111185).

#### 5.4.2. Microarray Data Processing

The microarray covered 24,123 probe sets representing 12,848 genes. The arrays passed the appropriate quality control criteria as previously proposed [59]. The data were GC-RMA normalized (Log2). In order to improve statistical power [60], uninformative data such as internal controls were discarded. Data were filtered by MAS5 (present rate > 50% per treatment group), standard deviation (comparison I: SD > 0.34; comparison II: SD > 0.19), and mean (m > 2.5) as the corresponding transcripts were unlikely to show altered mRNA abundances. In comparison I, the filtering revealed 9541 probe sets corresponding to 6640 genes (comparison II: 9079 probe sets corresponding to 6328 genes). Relative mRNA differences were analyzed using a linear model including treatment and glucose levels as fixed effects and cell culture passage as a random effect (SAS version 9.4; SAS Institute, Cary, NC, USA). To account for multiple testing, p-values were converted to a set of q-values [61]. The level of significance was set at *p* ≤ 0.05 and *q* ≤ 0.05, respectively. Lists of altered transcripts were evaluated using Ingenuity Pathway Analysis (IPA, Ingenuity Systems, Redwood City, CA, USA). The significance of association between the dataset and pathway was set at *p* ≤ 0.05. 

### 5.5. qPCR

Quantitative PCR (qPCR) was performed on nine genes identified from the analysis of the gene expression data as potential candidate genes for key mechanisms regulated by DON-treatment. The primers were designed from the same porcine expressed sequence tags (EST) used for the development of the respective probe sets on the Affymetrics GeneChip^®^ Porcine Genome Array (Table 4).

Each 1 µg of template RNA was subjected to reverse transcription with First Aid Reverse Transcription Reagents (Fermentas, Germany) essentially as described by the manufacturer with the supplied random hexamer primers in a ThermalCycler TC1 (Biometra, Germany). The resulting cDNA samples were used for qPCR amplification.

Quantitative PCR amplification was performed for all genes under the following conditions on an iCycler (BioRad, München, Germany): 1.5 min at 95 °C, 5 min at 95 °C followed by 40 cycles of 30 s at 95 °C and 60 s at an optimal primer annealing temperature (Table 4). Melting curve analysis (50–95 °C) was used for assessing amplification specificity. The reaction volume of 20 µL contained 10 µL Maxima Mastermix (2×, ThermoFisher [Fermentas], Waltham, MA, USA) with SYBR^®^ Green and Fluorescein as internal standards, 2 pmol/µL of the respective primers (3 µL each), 2 µL nuclease free water, and 2 µL cDNA (60 ng/µL). 

Analysis of the expression data was done according to the relative standard curve method. A standard curve was derived for each single gene from a serial dilution of the cDNA. The analysis comprised of five independent experiments with each sample in triplicate. The ddCt method [62] was used for the calculation of differences in the gene expression (ratio = 2^−ddCt^). The differences between the DON-treated samples and the untreated control in the relative quantification (rq) values were normalized to the individual expression of the two housekeeping genes β-actin and 18S.

### 5.6. Western Blot

For the protein analysis, IPEC-J2 was cultured in ThinCerts^TM^ with a 15 mm diameter. At least three independent experiments were performed. Medium was withdrawn, cells were washed in phosphate buffered saline (PBS), and sodium dodecyl sulfate (SDS) loading buffer was added (10 mL containing 600 µL 1 M Tris base pH 6.8, 1000 µL glycerol, 2000 µL 10%-SDS, 500 µL 0.1%-bromophenol blue, 500 µL β-mercaptoethanol; 5.4 mL aqua dest). For protein denaturation, the lysate was heated up to 95 °C for 5 min. Quantification of protein content was performed using Molecular probes^®^ Qubit Protein Assay Kit and Qubit^®^ 2.0 Fluorometer (both Thermo Fisher (Invitrogen], Waltham, MA, USA) in accordance to the manufacturer’s protocols. 

For Western blot, 40 µg of protein sample as well as Page Ruler^TM^ prestained protein ladder (SM0671; Thermo Fisher [Fermentas], Waltham, MA, USA) were placed on SDS polyacrylamide gel. After electrophoresis, samples were transferred to 0.45 µm PVDF membrane by semi-dry electroblotting using TRANS-BLOT^®^ SD Semi Dry Transfer Cell (Bio-Rad, Munich, Germany). Protein detection was performed employing the BM Chemiluminescence Western Blotting Kit (Mouse/Rabbit) by following the manufacturer’s instructions (Roche, Basel, Switzerland). Primary antibodies were utilized to identify specific proteins: rabbit anti-PHB (1:2000, o.n. 4 °C; Abcam, Cambridge, UK), rabbit anti-SLC7A11 (1:2000; o.n. 4 °C, antibody verify, Las Vegas, NV, USA), rabbit anti-TXNIP (1:2000; o.n. 4 °C; abcam, Cambridge, UK), and mouse anti-β-actin 1:40000 (Sigma-Aldrich, Munich, Germany). Blots were visualized by MultiImage^TM^ Light Cabinet (Alpha Innotech, Kasendorf, Germany).

### 5.7. Oxygen Measurement

The oxygen uptake was measured with a Microx TX3 (Presens, Regensburg, Germany). The sensor was calibrated as per the manufacturer’s instructions (manual; 2-point calibration). The oxygen sensor was placed directly over the cells/membrane, which were stored in an incubator at 39 °C (5% CO_2_ and 100% humidity). After 30 min of equilibration, the measurement was started. The oxygen content in the cell medium (apical) was measured and averaged over 10 min at intervals of 30 s. Inserts with medium but without cells were used as blank (the basic content of oxygen directly over the membrane). The oxygen uptake was given due to the difference between both values (blank − sample value = oxygen uptake). 

At the end of the experiment, cells were fixed with ethanol (96%; 30 min; 4 °C) and acetone (30 s). In the next step, cells were stained with 4’,6-Diamidin-2-phenylindol (5 min; 1:10 in 0.1 M phosphate buffer (PB). Next, cells were washed with 0.1 M PB and mounted with Mowiol. Membranes were used for cell counting. Therefore, five randomized pictures were taken from each membrane.

### 5.8. Lactate and Glucose Measurement

In order to measure the oxygen content within the cell culture medium, IPEC-J2 was grown on ThinCerts^TM^, but the medium was unaltered during the final 72 h of cultivation and treatment. After the termination of cell culture, the medium was fully withdrawn from the cells and transferred into separate tubes according to the compartment distinguishing between the upper (apical) and lower (basolateral) compartments. All samples were stored on ice until measurement. Cell-free cell culture medium was used as a blank. Glucose and lactate concentrations were determined immediately using Cobas C 501 (Roche, Basel, Switzerland) as well as the reagents of test systems GLUC2 and LACT2 (Roche, Basel, Switzerland), respectively. The differences in glucose and lactate concentration between the blank and samples were considered as glucose consumption and lactate production. Glucose measurements of DON-treated cells under low glucose conditions resulted in values which are located below the detection limit of 0.11 mmol/L. Therefore, analyses of experiments with low glucose were not shown in the results.

### 5.9. ATP Measurement

Cells were seeded on 12-well-ThinCerts for 10 days. On day 7, cells were treated apical or basolateral with different DON concentrations 50 ng/mL, 200 ng/mL, and 2000 ng/mL. On day 9, control cells were treated with or without carbonylcyanid-4-trifluormethoxyphenylhydrazon (FCCP, 5 µM in DMSO, Sigma-Aldrich, Hamburg, Germany), 2-Deoxy-glucose (2DG; 5 mM in glucose-free medium) or with medium without glucose for 24 h. On day 10, media were removed and a boiling hot puffer (300 µL/well; 100 mM TRIS; 4 mM EDTA; pH 7.75, Roth, Karlsruhe, Germany) was added to the cells. In the next step, cells were scraped off the membrane with a cell scraper. The cell suspension was transferred into a tube, incubated for 2 min at 100 °C, centrifuged at 1000× *g* for 60 s. Supernatants were pipetted into a 96-well-microplate (50 µL/well; triplicates; Greiner bio-one; Frickenhausen, Germany). Samples kept on ice until measurement. An ATP-standard curve was prepared following the manufacturer’s instructions (five readings within 5 min; 25 °C; ATP Bioluminescence Assay Kit CLS II; Roche, Basel, Switzerland).

### 5.10. Analyses of the Protein Biosynthesis

#### 5.10.1. Metabolic Labeling and Treatment of IPEC-J2

Labeling experiments were performed according to Müller et al. [63] in methionine-free DMEM medium supplemented with either 4 mM methionine (Met) or 4 mM azidohomoalanine (AHA). At day 7, cells were incubated with different DON-concentrations for 48 h with DMEM/HAMF12 (see above). 

Subsequently, media were exchanged to Met-free media and cells were pre-incubated for 30 min. Afterwards, the Met-free media were replaced by the above described media containing AHA or Met as a control. 

After 24 h cells were harvested with trypsin (10 min; 39 °C), centrifuged (10 min; 350× *g*), and washed with PBS. Subsequently, cell pellets were washed twice with PBS (pH 7.8) and frozen at –80 °C.

#### 5.10.2. Bioorthogonal Non-Canonical Amino Acid Tagging (BONCAT)

Tagging of AHA labeled proteins was performed as essentially described in Dieterich et al. and Landgraf et al. [64,65]. Briefly, cell pellets were lysed for 5 min at 95 °C in 300 µL 1× PBS, pH 7.8, containing 0.2% Triton X100, 0.1% SDS, 1× cOmplete^TM^ EDTA-free protease inhibitor cocktail (Roche, Basel, Switzerland), and 250 U/mL Benzonase^®^ nuclease. The resulting protein extracts were centrifuged for 5 min at 14,000× *g* at 4 °C, and the supernatant transferred into fresh 1.5 mL Eppendorf tubes. For click-chemistry, samples were supplemented with 0.2 mM Triazole ligand (tris[(1-benzyl-1*H*-1,2,3-triazol-4-yl) methyl] amine (TBTA), 25 µM biotin-PEO_3_-alkyne-tag, and 0.2 mg/mL copper(I)bromide-suspension (Acros/Thermo Fisher Scientific, Munich, Germany). After the addition of each reagent, samples were thoroughly vortexed for 15 s. Subsequently, samples were incubated under continuous agitation at RT. After 2 h reaction time, precipitates were removed by centrifugation for 5 min at 3000× *g*, 4 °C, and the remaining protein extracts were further processed for quantification, SDS-PAGE, and Western blot.

#### 5.10.3. Western Blot Experiments and Quantitative Analysis

For SDS-PAGE and Western blot analysis, protein fractions were solubilized with 4× SDS sample buffer (250 mM Tris–HCl, pH 6.8, 1% SDS, 40% glycerol, 20% β-mercaptoethanol, 0.004% bromophenol blue), boiled for 5 min (95 °C) and separated on 5–20% SDS–polyacrylamide gradient gels, subsequently followed by transfer onto nitro cellulose membranes. After blotting, membranes were blocked with blocking solution (5% dry milk, 0.1% Tween 20 in 1× TBS) for 1 h at RT with gentle agitation. Incubation with primary antibodies (anti-biotin, 1:10,000) was done overnight at 4 °C in blocking solution. After intensive washing, blots were incubated for 90 min at room temperature with HRP-conjugated secondary antibodies (1:10,000) in blocking solution and finally developed with ECL reagent (Thermo Fisher Scientific, Waltham, MA, USA) using the Odyssey^®^ Fc luminescence detector (LI-COR). 

For normalization, identical samples were separated by SDS-PAGE and stained for 1 h with 0.05% Coomassie brilliant blue dissolved in 50% methanol and 10% acetic acid, followed by destaining using a solution consisting of 5% methanol and 7% acetic acid. Determination and quantification of the Western blot signals and Coomassie-stained gels was done using the Image Studio Lite version 5.0 software from LI-COR.

#### 5.10.4. Statistical Analysis

Data were tested for normal distribution with the Kolmogorov–Smirnov test with SPSS 24. In the event of a normal distribution, a Levene test was used to test for variance homogeneity. Furthermore, an ANOVA (Levene Test negative; Tukey Test) or a Welch ANOVA (Levene Test positive; Games-Howell) was used for further analyses. In the case of a significant result (no normal distribution), a Kruskal–Wallis test was used to check for differences between groups. In the case of a significant result, a Mann–Whitney test was performed and p-values were corrected (alpha correction). qPCR data for TXNIP based on pairwise comparisons of high and low glucose (Figure 2B) were also checked for normal distribution with the Kolmogorov–Smirnov test (delta-CT values). In the event of normal distribution, a t-test was performed to display significant changes. A Mann–Whitney test was used in the event of no normal distribution. The same procedure was performed for SLC7A11 and PHB. 

## Figures and Tables

**Table 1:** Significantly regulated pathways. All genes were analyzed via DAVID and resulted in significantly regulated KEGG-pathways. All basically regulated (red marked in Figure 1) genes in the comparison of high and low glucose under impact of DON were examined.

Number	Pathways
1	Metabolic pathways
2	Pathways in cancer
3	Endocytosis
4	PI3K-Akt signaling pathway
5	Biosynthesis of antibiotics

**Table 2:** Analyzed genes via qPCR (*N* = 5). Different genes were analyzed with qPCR, which were related to metabolic pathways: TXNIP, COX5B, GLUT1, MCT1, SGLT1, PHB and SLC7A11. All comparisons were examined under low and high glucose. Therefore, 18S and actin were used as housekeeping genes. All comparisons were examined under low and high glucose. All treatment groups (50 ap; 50 bl; 200 ap; 200 bl, 2000 ap and 2000 bl) were compared to the control [CON = 100%] and significantly regulated genes were asterisked (*p* < 0.05 *; *p* < 0.001 **; *p* < 0.001 ***). The values shown in the table represent relative quantification [%].

	**TXNIP**			
	**18S**		**Actin**	
	**Low**	**High**	**Low**	**High**
**CON**	100	100	100	100
**50 ap**	109.68	112.25	138.83	128.94 *
**50 bl**	100.46	115.4	123.68	130.74 *
**200 ap**	86,65	113.29	113.81	130.13 *
**200 bl**	125.99	123.68	127.16	133.18 *
**2000 ap**	216.1 ***	127.75	110.33 *	142.08 *
**2000 bl**	111.03	124.26	96.82	94.17 ***
	**COX5B**			
	**18S**		**Actin**	
	**Low**	**High**	**Low**	**High**
**CON**	100	100	100	100
**50 ap**	102.34	89.09	129.53	102.45
**50 bl**	105.6	92.02	127.62	104.36
**200 ap**	85.86	96.37	112.77	110.82
**200 bl**	109.18	89.09	110.19	96.03
**2000 ap**	242 ***	144.47	125.12 ***	158.91 ***
**2000 bl**	172.4	140.03 ***	149.48	105.92
	**GLUT1**			
	**18S**		**Actin**	
	**Low**	**High**	**Low**	**High**
**CON**	100	100	100	100
**50 ap**	127.16	91.59	160.96 **	105.21
**50 bl**	126.58	97.72	155.83 **	110.7
**200 ap**	119.75	138.84	157.28 **	159.48 *
**200 bl**	150.18	115.4	151.57 *	124.26
**2000 ap**	460.01 ***	891.61 ***	237.84 ***	985.62 ***
**2000 bl**	688.67 ***	483.44 ***	591.46 ***	366.38 ***
	**MCT1**			
	**18S**		**Actin**	
	**Low**	**High**	**Low**	**High**
**CON**	100	100	100	100
**50 ap**	115.94	85.46	146.75 ***	94.95
**50 bl**	119.27	88.27	145.4 ***	96.72
**200 ap**	93.74	92.99	123.11	104.56
**200 bl**	99.08	88.27	100	91.93
**2000 ap**	194.08 *	112.8	99.66	125.58
**2000 bl**	154.3	100	133.79 **	73.3
	**SGLT1**			
	**18S**		**Actin**	
	**Low**	**High**	**Low**	**High**
**CON**	100	100	100	100
**50 ap**	118.65	107.14	150.18 ***	123.32
**50 bl**	123.11	114.87	151.57 ***	130.13
**200 ap**	88.68	80.8	116.47	92.08
**200 bl**	118.41	108.21	117.98	118.53
**2000 ap**	176.23 **	78.43	85.75	90.27
**2000 bl**	81.78	62.2	67.43 ***	46.12 ***
	**PHB**			
	**18S**		**β-actin**	
	**Low**	**High**	**Low**	**High**
**CON**	100	100	100	100
**50 ap**	127.16	91.59	160.96	105.21
**50 bl**	126.58	97.72	155.83	110.7
**200 ap**	119.75	138.84	157.28	159.48
**200 bl**	150.18	115.4	151.57	124.26
**2000 ap**	460.01 ***	891.61 ***	237.84 ***	985.62 ***
**2000 bl**	688.67 ***	483.439 ***	591.46 ***	366.38 ***
	**SLC7A11**			
	**18S**		**β-actin**	
	**Low**	**High**	**Low**	**High**
**CON**	100	100	100	100
**50 ap**	127.75	110.19	161.7	126.58
**50 bl**	126.99	136.29	153.48	154.4 ***
**200 ap**	92.87	84.67	121.98	97.27
**200 bl**	97.72	108.17	98.62	116.47
**2000 ap**	30.57 ***	62.31	15.8 ***	68.87 **
**2000 bl**	31.59 ***	92.02	28.32 ***	69.74

**Table 3:** Significantly regulated pathways under low glucose conditions. A total of 186 genes (red marked in Figure 3) that were significantly regulated in the comparisons between the control and DON treatment groups under low glucose were analyzed. The analyses with DAVID resulted in significantly regulated KEGG-pathways.

Number	Pathways
1	Spliceosomes
2	RNA transport
3	Epstein–Barr virus infection
4	Ribosome biogenesis in eukaryotes
5	mRNA surveillance pathway

**Table 4:** Used primer pairs.

Gene	Left (5′-3′)	Right (5′-3′)	Product Size	Temperature [°C]
18S	GCAATTATTCCCCATGAACG	GGCCTCACTAAACCATCCAA	123	56.5
beta-actin	GATGAGATTGGCATGGCTTT	CACCTTCACCGTTCCAGTTT	122	58.3
TXNIP	AGCAGCCAAGAGAACAGAGA	TCCACGGACACAATACCCA	118	57.2
PHB	TGA AAA CTC TGC CCC TGT GA	TCT GCA GGA CTC ACA TCT CG	119	57.3
SLC7A11	TAA ATT TGG GTG CAA TGT GAT GT	TTG AAG CAA CTA GAA GCA TGA CA	99	54.9
MCT1	TCCATCATGTTGGCTGTCAT	GAAGGAAGCTGCAATCAAGC	129	58.5
COX5B	GGAGAGGGAGGTCATGATGG	CCACTATCCGCTTGTTGGTG	128	59.4
SGLT-1	AAGCTGGTCATGGAGCTGAT	AGACGTCCATGGTGAAGAGG	127	61.5
GLUT1	GAGCCCTGCCTAGACACTTG	CCACCTCTTGGGGTAGAAGA	112	62

## Supplementary Materials

The following are available online at http://www.mdpi.com/2072-6651/10/11/464/s1, Figure S1: TEER measurements under high (**A**) and low (**B**) glucose conditions.

## Abbreviations

AcCoA	acetyl coenzyme A
AHA	azidohomoalanine
ap	apical
ATP	adenosinetriphosphate
bl	basolateral
BONCAT	bioorthogonal non-canonical amino acid tagging
CAV2	caveolin-2
COX5B	cytochrome C oxidase subunit 5B
CYP26B1	cytochrome P450 26B1
2DG	2-deoxy-glucose
DON	deoxynivalenol
EGF	epidermal growth factor
EST	expressed sequence tags
ETC	electron transport chain
FADH	flavine adenine dinucleotide
FBS	fetal bovine serum
FCCP	carbonylcyanid-4-trifluormethoxyphenylhydrazon
GAPDH	glycerinaldehyd-3-phosphate dehydrogenase
GSH	glutathione
HEPES	4-(2-hydroxyethyl)-1-piperazine ethanesulfonic acid
IPEC-J2	intestinal porcine epithelial cells
ITS	insulin-transferrin-selenium
Met	methionine
MEST	mesoderm-specific transcript homologues protein
NADPH	nicotinamide adenine dinucleotide phosphate
OxPhos	oxidative phosphorylation
PB	phosphate buffer
PBS	phosphate buffered saline
PHB	prohibitin
PKR	protein kinase R
PVDF	polyvinylidenfluoride
ROS	reactive oxygen species
RQ	relative quantification
SDS	sodium dodecyl sulfate
SLC6A19	system B(0) neutral amino acid transporter AT1
SLC7A11	solute carrier family 7 member 11
TAGLN	transgelin
TBTA	tris[(1-benzyl-1*H*-1,2,3-triazol-4-yl)methyl]amine
TRA1	tumor rejection antigen 1 (≙ GRP94, HSP90)
TRIS	tris(hydroxymethyl)aminomethane
VDUP1	vitamin D3 upregulated protein1
