# Supplementary material for: Deoxynivalenol Affects Cell Metabolism and Increases Protein Biosynthesis in Intestinal Porcine Epithelial Cells (IPEC-J2): DON Increases Protein Biosynthesis
Source: Toxins (Basel). 2018 Nov 9;10(11):464. doi: 10.3390/toxins10110464 (PMC6266275; doi:10.3390/toxins10110464)
Supplement: Supplementary file 1 [file toxins-10-00464-s001.pdf]

Toxins **2018**, *10*, 464; doi:10.3390/toxins10110464

Supplementary Materials: Deoxynivalenol Affects Cell Metabolism and Increases Protein Biosynthesis in Intestinal Porcine Epithelial Cells (IPEC-J2)

DON Increases Protein Biosynthesis

Constanze Nossol, Peter Landgraf, Stefan Kahlert, Michael Oster, Berend Isermann, Daniela C. Dieterich, Klaus Wimmers, Sven Dänicke and Hermann-Josef Rothkötter

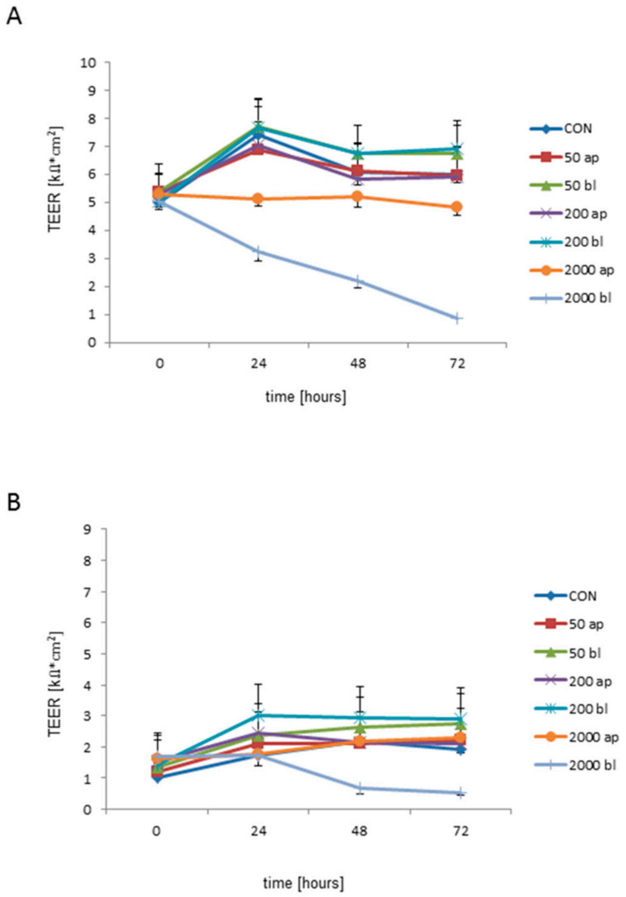

Figure S1. TEER measurements under high (**A**) and low (**B**) glucose conditions.
